# Spleen Transient Elastography and Damping Index Identify a Subgroup of Patients Without an Acute or Chronic Response to Beta-Blockers

**DOI:** 10.3389/fmed.2022.900073

**Published:** 2022-06-22

**Authors:** Elba Llop, Christie Perelló, Teresa Fontanilla, Juan de la Revilla, Marta Hernández Conde, Marta López, Javier Minaya, Carlos Ferre, Javier Abad, Carlos Fernández Carrillo, José Luís Martínez, Natalia Fernández Puga, María Trapero, Ismael El Hajra, Elena Santos, José Luis Calleja

**Affiliations:** ^1^Department of Gastroenterology and Hepatology, Hospital Universitario Puerta de Hierro Majadahonda, Madrid, Spain; ^2^Instituto de Investigación Sanitaria Puerta Hierro-Segovia Arana (IDIPHISA), Madrid, Spain; ^3^CIBERHD, Madrid, Spain; ^4^Department of Radiology, Hospital Universitario Puerta de Hierro Majadahonda, Madrid, Spain

**Keywords:** spleen stiffness, damping index, chronic response to betablockers, acute response to betablockers, transient elastography

## Abstract

**Background and Aims:**

Monitoring of acute or chronic response to beta-blockers in patients with liver cirrhosis is based on the measurement of the HVPG. Our aim was to evaluate the response to beta-blockers with non-invasive techniques.

**Patients and Methods:**

This is a prospective observational study. Consecutive patients with an indication of primary or secondary prophylaxis of variceal bleeding who did not meet exclusion criteria were included. Acute response and chronic response were evaluated. Baseline and after acute and chronic response hepatosplenic measurements of TE and ARFI were obtained. Contrast-enhanced Doppler ultrasound was performed before and after acute and chronic responses.

**Results:**

From June 2015 to May 2018, 55 patients (14 with exclusion criteria) were included. We analyzed 41 patients, mean age 57 (*SD*: 8), 82.9% men, alcohol 43.9%, children A/B/C 78%/17.1%/4.9%, and 87.8% on primary prophylaxis. In all, the acute response was performed and was positive in 68.3% (CI 95: 55–85%). The chronic response was performed in 30 (73.2%) and was positive in 36.7% (CI 95: 18–55%). Basal measurements significantly related to acute response were spleen TE [responders 58.4 (*SD*: 23.0) KPa vs. non-responders 75 (*SD*: 0) KPa; *p* = 0.02] and damping index [non-responders 0.96 (0.8) vs. responders 0.44 (0.4), *p* = 0.01], and with chronic response, the spleen TE [responders 58.1 (*SD*: 21.4) KPa vs. non-responders 73.2 (*SD*: 5.5) KPa; *p* = 0.02], and damping index [non-chronic responders 0.8 (0.7) vs. chronic responders 0.4 (0.4), *p* = 0.04]. A spleen TE ≥ 74 KPa had a high sensitivity of 100% and specificity of 60% and a high NPV100% for predicting poor acute response to beta-blockers. The damping index > 0.6 showed moderate sensitivity of 67% and specificity of 69% with a high NPV of 82% for predicting poor acute response to beta-blockers. The combination of both measurements for predicting poor acute response to beta-blockers had an AUC of 0.8 (CI 95: 0.5–0.9). A spleen TE ≥ 74 KPa had a high sensitivity of 87% and specificity of 71% with a high NPV of 71% for predicting poor chronic response to beta-blockers. A damping index > 0.6 had moderate sensitivity of 60%, specificity of 82%, and NPV of 56% for predicting poor chronic response to beta-blockers. The combination of both measurements for predicting poor chronic response to beta-blockers had an AUC of 0.8 (CI 95: 0.7–0.9).

**Conclusion:**

Spleen TE and damping index can identify a subgroup of patients with poor acute or chronic response to beta-blockers.

## Introduction

Acute variceal bleeding is one of the main complications of portal hypertension in liver cirrhosis (1). Approximately 15–20% of initial bleeding episodes can be fatal (2), and after a first bleeding episode, the probability of survival at 1 year is reduced to 50% (3). Chronic response to beta-blockers by reducing the HVPG by more than 20% with respect to the baseline value or below 12 mmHg significantly reduces the risk of hemorrhagic recurrence (4). This hemodynamic response is evaluated 1–3 months after starting treatment, which requires the performance of two hepatic vein catheterizations. To avoid these studies, the utility of the acute hemodynamic response to beta-blockers has been evaluated (decrease in HVPG ≥ 10%) (5). Several studies have confirmed that the acute hemodynamic response maintains the same predictive capacity for gastrointestinal bleeding and long-term complications as the classic chronic hemodynamic response (6–10). However, hepatic vein catheterization is not available in most centers and has the disadvantage of being an invasive method. For this reason, the beta-blocker dose is usually adjusted based on clinical parameters such as heart rate, blood pressure, and tolerance to treatment (4). However, there is no correlation between the efficacy of the treatment and these clinical parameters (4).

In the past 10 years, non-invasive methods have been developed to predict the existence of CSPH and esophagogastric varices (11). Liver TE has shown a good correlation with HVPG (12–15), being able to diagnose CSPH (AUROC = 0.921 for low cutoff values of 13.6–18 kPa) (15). Other studies have concluded that liver stiffness values < 13.6 kPa are considered valuable to rule out CSPH (sensitivity > 90–95%), while LS > 22 kPa appears to accurately predict it (specificity > 90–95%) (16). Moreover, other liver elastographic techniques (ARFI, 2D-SWE, or MR) have been evaluated to predict CSPH, showing promising results (17–19). However, the correlation between these methods is good for values lower than 12 mmHg but poor in more advanced cirrhosis stages. Probably, because in these stages, other factors such as increased portal flow secondary due to splanchnic vasodilation and hyperdynamic circulation play a key role in HVPG (20). Thus, to try to evaluate the splanchnic dynamic component, spleen elastography (by TE, ARFI, 2D-SWE, or MR) has been developed (21–26). Spleen elastography has shown a good correlation with HVPG (22). Different cutoff points have been proposed to detect CSPH (27).

The application of these non-invasive methods to assess the hemodynamic response to beta-blockers has not yet been sufficiently evaluated. Studies regarding the non-invasive evaluation of the acute or chronic response to beta-blockers are inconsistent. Beta-blocker treatment reduced HVPG without being able to detect significant changes in liver elastography, possibly because vasoconstrictor drugs act on the extrahepatic dynamic component of portal hypertension and do not influence the intrahepatic vascular component, which is the one evaluated with liver elastography (28). In another study, changes in spleen stiffness measured by ARFI exhibited good performance in predicting hemodynamic response to NSBB prophylaxis in patients with high-risk esophageal varices (29). In addition, in a study using TE, changes in spleen stiffness after NSBB initiation correlated with changes in HVPG (*r* = 0.784), and spleen stiffness presented excellent accuracy for the identification of responders (AUROC = 0.973) (30). However, these results have not been confirmed and more studies are needed to investigate the potential role of liver and spleen stiffness in predicting the acute and chronic response to beta-blockers.

Finally, abdominal Doppler ultrasound has also shown its usefulness in predicting portal hypertension. In fact, multiple indices have shown a good correlation with HVPG and CSPH (31, 32). Of these, the damping index of the hepatic vein wave form (calculated by dividing minimum velocity by maximum velocity of hepatic vein waveform) has shown a good correlation with the HVPG and a value higher than 0.6 determines the presence of HVPG > 12 mmHg (33). In this study, the damping index showed a good correlation with HVPG in beta-blocker responders even though the sample size was small (33). In contrast, contrast-enhanced ultrasound evaluating the hepatic vein transit time has also shown a correlation with portal hypertension and esophagogastric varices (34). Again, these results have not been confirmed, and more studies are needed to confirm the role of damping index and hepatic vein transit time in predicting the acute and chronic response to beta-blockers.

Therefore, the aim of our study was to evaluate different non-invasive techniques to predict the acute and chronic response to beta-blockers.

## Patients and Methods

This is a prospective observational study. Consecutive patients with liver cirrhosis, aged between 18 and 75 years and of both sexes, who indicated beta-blockers as primary or secondary prophylaxis of gastrointestinal bleeding due to esophageal varices (according to Baveno VI recommendations) (35) were included. The diagnosis of cirrhosis was established by liver biopsy or by the existence of compatible clinical and radiological criteria. Patients who met any of the following criteria were excluded: complete portal vein thrombosis, active alcohol consumption (abstinence from alcohol for at least 6 months), active viral hepatitis (properly treated for HCV and HBV before inclusion), contraindication for beta-blockers, hepatocellular carcinoma that did not meet Milan criteria, cholestatic liver disease, HIV coinfection, allergy to iodized contrast, pregnancy, concomitant consumption of other drugs that could modify portal pressure (nitrates, prazosin), and refusal to sign the informed consent.

### Beta-Blocker Administration

The beta-blocker used was carvedilol for primary prophylaxis and propranolol for secondary prophylaxis. The first dose was administered 24 h after the baseline hemodynamic study. The dose was progressively increased until reaching a heart rate of not less than 55 bpm or systolic blood pressure of not less than 90 mmHg or the maximum dose of beta-blocker (25 mg every 24 h of carvedilol/160 mg every 12 h of propranolol) or the appearance of limiting symptoms. All patients must reach the maximum tolerable dose within 1 month of the first hemodynamic study.

### Hemodynamic Study

The examination was carried out after an 8-h fast. Before each study, the pressure transducer was calibrated according to the usual procedure. During the entire procedure, electrocardiographic, pulse oximetry, blood pressure, and heart rate monitoring were performed using a constant monitor (Dash 2500, GE Healthcare, Freiburg, GE). All hemodynamic measurements were recorded in digital format through a multichannel system for later analysis (Power-Lab 4/30, Lab-Chart 7, AD instruments, Chalgrove, United Kingdom). After placing an 8F introducer catheter (Cook Medical, Bloomington, United States) under local anesthesia and using the Seldinger technique in the right internal jugular vein, a 7F balloon catheter (Edwards Lifesciences, Irvine, United States) was inserted under fluoroscopic control to the right hepatic vein. Measurements of FSHP and WSHP after inflating the distal balloon were obtained. Correct venous occlusion was confirmed by fluoroscopic control after injection of a small amount of iodized contrast. Each WSHP measurement was repeated in triplicate with a minimum recording time of 2 min. Each FSHP measurement was performed at < 2 cm from *de inferior* cava vein and during 15 s. HVPG was calculated by obtaining the difference between WSHP and FSHP. Right atrial pressure and inferior vein cava pressure were measured as well.

Evaluation of the acute response to propranolol: After obtaining all baseline hemodynamic measurements and without removing the introducer, endovenous propranolol (0.15 mg/kg) was administered for 10 min. HVPG was calculated again 10 min after the infusion, following the methodology previously described (5). An acute response was considered positive if it was greater than 10% compared to baseline.

Evaluation of the chronic response to propranolol: A second hemodynamic study was repeated to evaluate the chronic response to beta-blockers in those patients who were correctly treated. The chronic response was defined as a reduction in HVPG greater than 20% compared to baseline or a reduction below 12 mmHg.

### Elastography Methods

Elastography methods were performed the same day before the baseline hemodynamic study and after evaluating the acute and chronic response to beta-blockers. A minimum of 6 h of fasting was required either for TE or ARFI.

### Hepatosplenic Transient Elastography

Hepatic TE was performed using FibroScan^®^ (Echosens, Paris, FR). It was performed with the patient in the supine position, with the right arm in hyperextension. The probe was placed between the ribs at the level of the right hepatic lobe, avoiding areas with artifacts or vessels, and 10 valid measurements were obtained. TE was considered to meet the reliability criteria when the success rate was equal to or greater than 60% and the interquartile range was less than 30%. Spleen TE was performed with the patient in a supine position, with the left arm in hyperextension. The transducer was placed in a left intercostal space chosen by ultrasound, avoiding the presence of significant vessels. Hepatic TE reliability criteria were applied. The results were expressed in KPa. Due to the fact that the start of the study was in 2015, the specific splenic probe that would have offered a greater range of values could not be used.

### Hepatosplenic Acoustic Radiation Force Impulse

Acoustic radiation force impulse was performed during the ultrasound examination using Siemens S2000 equipment, with a 4C1 convex probe and the specific software for ARFI Virtual Touch, from Siemens. The measurement was taken with the patient in a supine position, with the right arm in hyperextension. An area in the right hepatic lobe located 2–3 cm below the hepatic capsule, free of large vessels or biliary structures, was chosen, and the measurements were made with the patient in apnea. Ten measurements were obtained, with the average being considered the representative value. The results were expressed in m/s. The spleen elastography measurement was performed with the patient in the supine position, with the left arm in hyperextension and apnea. The measurement was carried out by placing the probe in an intercostal space in which the splenic parenchyma was free of large vessels and 1 cm below the splenic capsule. Ten measurements were required with the same quality criteria as the hepatic ARFI. The operator in charge of its performance was a sonographer with extensive experience in the technique who was blind to the results of the hemodynamic studies and the TE. TE reliability criteria were used for both liver and spleen ARFI since they had also been shown to be useful with ARFI (36).

### Abdominal Doppler Ultrasound With Contrast

Abdominal Doppler ultrasound with contrast was performed in all patients before and after the evaluation of acute and chronic responses. A minimum of 6 h of fasting was required. During Doppler ultrasound, a measurement of the longitudinal diameter of the spleen was made, and hemodynamic parameters of the portal vein and the suprahepatic vein were obtained, including portal vein diameter, portal vein speed, damping index, and contrast-enhanced ultrasound evaluating the hepatic transit time as previously described (33, 34).

### Statistical Analysis

Continuous variables were described as the mean and standard deviation. Differences between means were analyzed using Student’s *t*-test. Categorical variables were described as numbers and percentages, and 95% CIs were given when necessary. Differences between proportions were analyzed using the Chi-squared test or Fisher’s exact test when appropriate. The Pearson correlation coefficient or Spearman coefficient were used to analyze the correlation between continuous variables. Receiver operating characteristic curves (ROC) were performed to evaluate the accuracy of non-invasive measurements for diagnosing poor response to beta-blockers. The Youden index was used to detect the best cutoff points of non-invasive measurements to detect poor response to beta-blockers. All statistical analyses were conducted using the statistics program Stata/IC 16.1.

## Results

**FIGURE 1:**
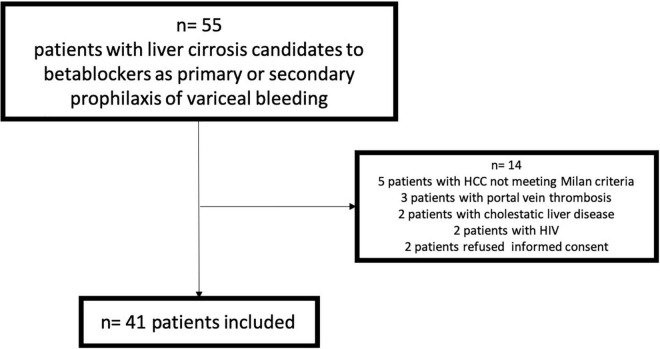
Flow chart of the study.

From June 2015 to May 2018, 55 patients were evaluated (14 with exclusion criteria) (Figure 1), And 41 were finally included. Baseline characteristics are described in Table 1. The acute response was performed in all the patients, and 28 [68.3% (CI: 55–85%)] showed acute response to beta-blockers. The chronic response was performed in 30 patients (73.2%) (6 patients denied informed consent for the second hemodynamic study, 4 showed intolerance to beta-blockers, and 1 died before the performance of the second hemodynamic study), and 11 [36.7% (CI: 18–55%)] showed chronic response to beta-blockers. Non-invasive and invasive measurements from baseline and after the acute and chronic response evaluation to beta-blockers are shown in Table 2.

**TABLE 1:** Baseline characteristics of the patients.

**Age (y)**	57 (8)
Sex (M:F)*	34:7 (82.9:17.1)
Etiology*	
Hepatitis C virus Alcohol Autoinmune Non-alcoholic fatty liver disease Other	11 (26.8) 18 (43.9) 4 (9.8) 5 (12.2) 3 (7.32)
Child pugh*	
A B C	32 (78.1) 7 (17.1) 2 (4.9)
Primary prophilaxis	87.8
MELD*	10.6 (0.6)
Laboratory test*	
Leucocites (cels*10^9^L) Hemoglobine (mg/dL) Platelets (cels*10^9^L) Creatinine (mg/dL) Sodium (mg/dL) Potasium (mg/dL) Bilirrubine (mg/dL) Albumine (mg/dL) ALT (U/**) AST (U/L)** Alkaline phosphatase (U/L) GGT (U/L)** INR**	6.2 (0.5) 12.8 (0.5) 114.0 (7.8) 0.8 (0.03) 139.6 (0.6) 4.3 (0.1) 1.6 (0.3) 3.9 (0.1) 41.1 (5.2) 51.2 (5.6) 134.2 (17.8) 121.8 (15.7) 1.3 (0.1)

*Quantitative data are given by mean and standard deviation, and qualitative data are given by number and percentage. **ALT, alanine aminotransferase; AST, aspartate aminotransferase; GGT, gamma-glutamyl transferase; INR, international normalized ratio.

**TABLE 2:** Baseline and follow-up invasive and non-invasive measurements after acute and chronic response to the beta-blockers evaluation.

	Baseline measurements (*n* = 41)	Acute response evaluation (*n* = 41)		Chronic response evaluation (*n* = 30)	
			
		Non-responders (*n* = 13)	Responders (*n* = 28)	Non-responders (*n* = 19)	Responders (*n* = 11)
Righ atrial pressure (mmHg)*	3.9 (2.6)	5.0 (3.5)	6.9 (2.7)	3.1 (2.2)	6.4 (2.8)
Inferior cava vein pressure (mmHg)*	7.5 (3.5)	6.8 (3.9)	8.6 (3.1)	6.4 (2.9)	9.9 (2.7)
FSHV (mmHg)*	9.2 (4.7)	9.8 (4.2)	10 (4.4)	6.7 (3.5)	15.7 (9.7)
WSHP (mmHg)*	25.9 (6.6)	27.9 (4.5)	23.3 (6.5)	25.3 (4.4)	24.1 (8.7)
HVPG (mmHg)*	17.0 (6.1)	18 (3.9)	13.1 (5.1)	18.6 (4.3)	8.8 (4.8)
Sistolic/Diastolic blood pressure (mmHg)*	136.3/76.3 (24.5/12.7)	147.1/78.4 (30.2/15.2)	136.2/79 (38.7/14.9)	116.4/66 (15.7/10.9)	133.6/75.6 (22.2/11.6)
Heart rate (bpm)*	74.3 (9)	64.3 (8.5)	61.0 (7.5)	62.8 (5.7)	61 (5.9)
Liver TE (KPa)*	38.3 (18.6)	34.7 (21.9)	33.3 (25.4)	33.3 (20.7)	34.3 (26.8)
Spleen TE (KPa)*	65.0 (19.4)	75 (0)	66.3 (10.7)	75 (0)	66.6 (15.6)
Liver ARFI (m/s)*	2.7 (0.7)	2.6 (0.5)	2.5 (0.7)	2.8 (0.7)	2.4 (0.5)
Spleen ARFI (m/s)*	3.3 (0.5)	3.4 (0.6)	3.2 (0.6)	3.3 (0.4)	3.5 (0.4)
Portal vein diameter (mm)*	13.1 (2.6)	13.3 (4.8)	12.8 (4.1)	13.7 (3.0)	12.3 (3.9)
Portal vein speed (cm/s)*	19.3 (5.4)	17.9 (3.4)	19.1 (4.3)	19.2 (6.1)	17.6 (2.9)
Hepatic artery speed (cm/s)*	74.2 (40.0)	64.5 (23.9)	69.4 (20.7)	58.2 (29.3)	54.8 (27.5)
Resistance index hepatic artery*	0.7 (0.2)	0.7 (0.1)	0.7 (0.1)	0.7 (0.1)	0.7 (0.6)
Spleen diameter (cm)*	14 (2.3)	16.4 (3.6)	13.7 (2.2)	14.3 (3.0)	14.5 (3.3)
Hepatic vein transit time (s)*	21.2 (7.1)	25.1 (5.0)	23.9 (4.9)	26.1 (8.6)	26.8 (10.3)
Damping index*	0.7 (0.6)	0.9 (0.8)	0.4 (0.4)	0.4 (0.3)	0.3 (0.3)

*Quantitative data are given by mean and standard deviation, and qualitative data are given by number and percentage.

### Correlation Between Hepatic Venous Pressure Gradient and Non-invasive Measurements

We evaluated the correlation between baseline HVPG and non-invasive measurements (Supplementary Table 1). Non-invasive measurements that showed moderate correlation with HVPG were liver TE (*r* = 0.5; *p* = 0.01), spleen TE (*r* = 0.3; *p* = 0.04), liver ARFI (*r* = 0.4; *p* = 0.02), spleen ARFI (*r* = 0.5; *p* < 0.01), and portal vein diameter (*r* = 0.4; *p* = 0.02). The damping index showed a low correlation with HVPG (*r* = 0.2; *p* = 0.04).

### Evaluation of Non-invasive Test for Prediction of the Acute Response to Beta-Blockers

We evaluated basal factors related to acute response to beta-blockers. We compared clinical baseline characteristics between responders and non-responders (Supplementary Table 2), and none of them were related to acute response to beta-blockers. Baseline non-invasive measurements related to non-response to acute response to beta-blockers were spleen TE, spleen diameter, and damping index (Table 3). We analyzed the change in non-invasive measurements before and after acute response measurement between acute responders and acute non-responders, and none of them were significantly related to the acute response to beta-blockers (Supplementary Table 3).

**TABLE 3:** Univariate analysis among baseline invasive and non-invasive measurements between acute responders and non-responders to beta-blockers.

	Acute non-response to beta-blockers (*n* = 13)	Acute response to beta-blockers (*n* = 28)	*P*	Chronic non-response to beta-blockers (*n* = 19)	Chronic response to beta-blockers (*n* = 11)	*P*
Righ atrial pressure (mmHg)*	4.3 (2.7)	3.1 (2.2)	0.2	3.1 (2.1)	3.9 (0.8)	0.4
Inferior cava vein pressure (mmHg)*	7.9 (4.0)	6.3 (2.9)	0.2	6.8 (2.7)	7.7 (4.5)	0.5
FSHV (mmHg)*	10.0 (5.4)	9.2 (4.4)	0.7	7.0 (4.8)	11.1 (5.7)	0.1
WSHP (mmHg)*	28.4 (5.4)	25.0 (7.0)	0.1	26.8 (4.5)	26.5 (8.6)	0.9
HVPG (mmHg)*	18.5 (4.8)	16.2 (6.7)	0.3	19.4 (5.7)	15.4 (5.3)	0.06
Sistolic/Diastolic blood pressure (mmHg)*	141.2 (24.2)/78.8 (13.5)	135.1 (24.8)/76.0 (12.0)	0.5	132.7 (22.5)/74.8 (8.3	142.1 (28.9)/74.4 (7.9)	0.3
Heart rate (bpm)*	74.0 (9.8)	74.0 (7.2)	0.9	74.8 (8.3)	74.4 (7.9)	0.9
Hepatic TE (KPa)*	36.3 (17.7)	38.9 (19.8)	0.8	43.0 (17.6)	31.8 (12.4)	0.2
Spleen TE (KPa)*	75 (0)	58.4 (23.0)	0.04	73.2 (5.5)	58.1 (21.4)	0.02
Hepatic ARFI (m/s)*	2.9 (0.4)	2.6 (0.8)	0.3	2.9 (0.6)	2.4 (0.8)	0.1
Spleen ARFI (m/s)*	3.5 (0.5)	3.3 (0.4)	0.2	3.4 (0.4)	3.5 (0.4)	0.5
Portal vein diameter (mm)*	13.2 (3.7)	12.8 (2.1)	0.7	13.5 (2.7)	12.7 (2.6)	0.5
Portal vein speed (cm/s)*	16.9 (5.0)	20.0 (5.1)	0.1	19.2 (6.3)	20.1 (5.1)	0.7
Hepatic artery speed (cm/s)*	62.9 (28.1)	81.8 (46.9)	0.2	68.2 (28.5)	72.8 (31.6)	0.7
Resistance index hepatic artery*	0.8 (0.1)	0.7 (0.2)	0.5	0.8 (0.2)	0.7 (0.1)	0.4
Spleen diameter (cm)*	16.2 (3.4)	13.7 (2.0)	0.01	14.5 (2.7)	14.3 (2.9)	0.9
Hepatic vein transit time (s)*	22.2 (6.2)	20.8 (7.7)	0.6	20.2 (6.5)	21.5 (10.4)	0.7
Damping index*	0.96 (0.8)	0.44 (0.4)	0.01	0.8 (0.7)	0.4 (0.4)	0.04
Damping index (>0.6)*	53.8	25	0.06	47.4	18.2	0.06

*Quantitative data are given by mean and standard deviation, and qualitative data are given by number and percentage.

**FIGURE 2:**
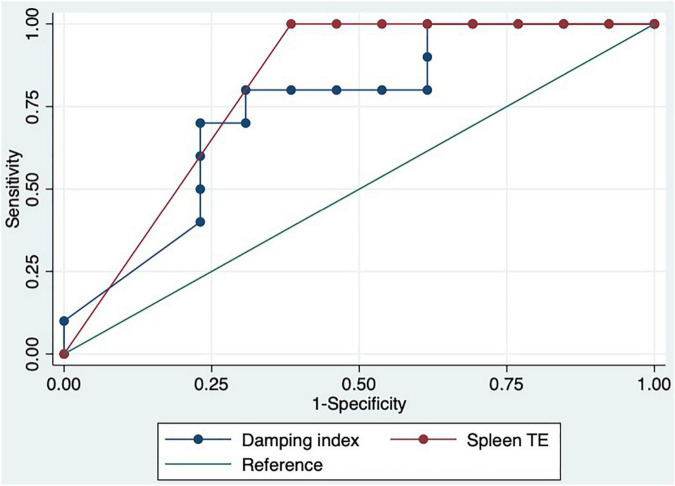
ROC curves of spleen TE and damping index to predict acute response to beta-blockers.

**FIGURE 3:**
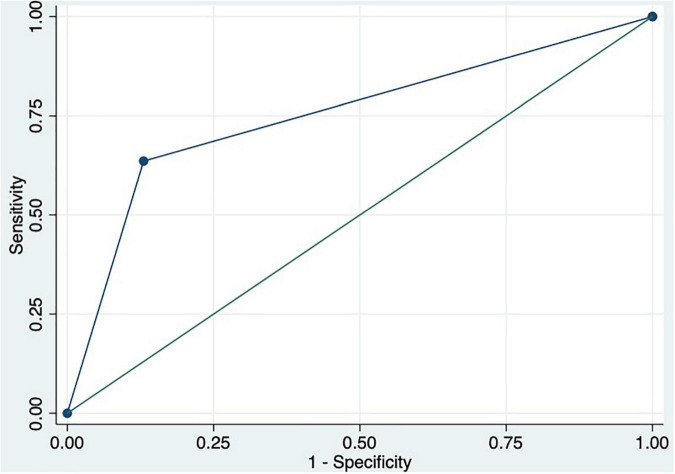
ROC curve of the combination of spleen TE ≥ 74 KPa and damping index > 0.6 to predict acute response to beta-blockers.

We performed a ROC curve analysis for estimating poor acute response to beta-blockers. The areas under the curve for spleen TE and damping index for predicting poor response to beta-blockers were 0.8 (CI: 0.7–0.9) and 0.7 (CI: 0.6–0.9), respectively (Figure 2). The best cutoff point of spleen TE to predict acute poor response to beta-blockers was ≥ 74 KPa (sensitivity: 100%; specificity: 60%; PPV: 63%; NPV: 100%; LHR + : 63%; LHR-: 0%). The best cutoff point of the damping index was > 0.6 (sensitivity: 67%; specificity: 69%; PPV: 50%; NPV: 82%; LHR + : 50%; LHR-: 25%). The acute response was not observed in 70% vs. 30%, *p* = 0.01 patients with spleen TE ≥ 74 KPa and damping index > 0.6. The combination of both measurements for predicting poor acute response to beta-blockers has an AUC of 0.8 (0.5–0.9) (Figure 3). Out of 41 patients, 10 had spleen TE ≥ 74 KPa and a damping index > 0.6.

### Predictors of Chronic Response to Beta-Blockers

We evaluated basal factors related to chronic response to beta-blockers. None of the baseline clinical characteristics were related to chronic response to beta-blockers. Among the non-invasive measurements, only spleen TE and damping index were related to chronic response to beta-blockers (Table 3). We analyzed the change in non-invasive measurements before and after chronic response measurement between chronic responders and chronic non-responders, and none of them was significantly related to the chronic response to beta-blockers (Supplementary Table 4).

**FIGURE 4:**
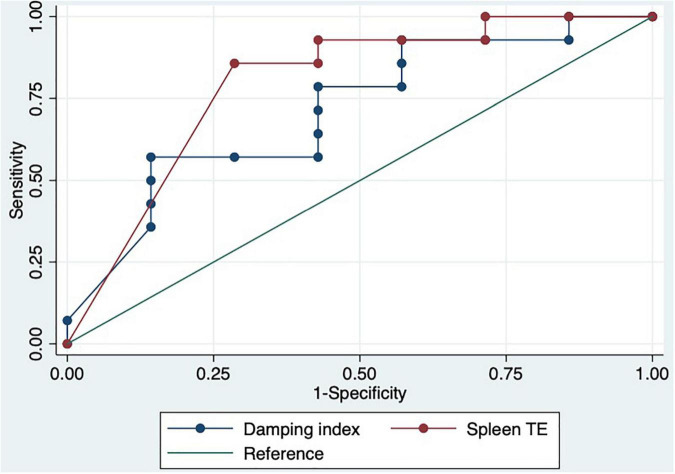
ROC curves of spleen TE and damping index to predict chronic response to beta-blockers.

**FIGURE 5:**
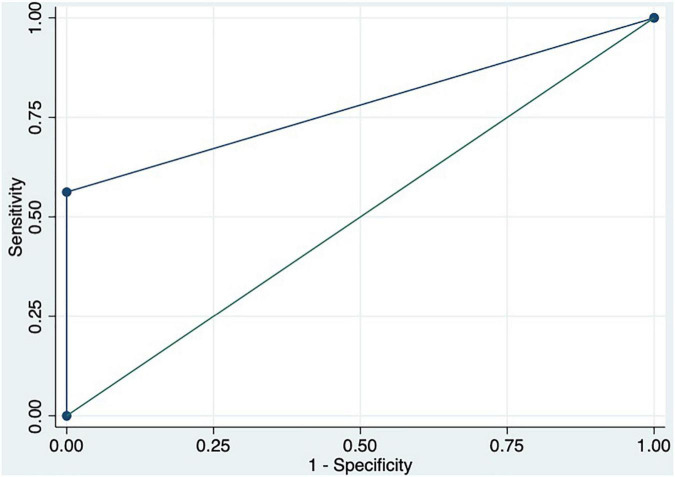
ROC curve of the combination of spleen TE ≥ 74 KPa and damping index > 0.6 to predict chronic response to beta-blockers.

We performed a ROC curve analysis for estimating poor acute response to beta-blockers. The AUC for spleen TE and damping index for predicting poor response to beta-blockers were 0.8 (CI: 0.6–0.9) and 0.7 (CI: 0.5–0.9), respectively (Figure 4). The best cutoff point of spleen TE to predict chronic poor response to beta-blockers was ≥ 74 KPa (sensitivity: 87%; specificity: 71%; PPV: 87%; NPV: 71%; LHR + : 87%; LHR-: 6%). The best cutoff point of the damping index was > 0.6 (sensitivity: 60%; specificity: 82%: predictive positive value: 83%; predictive negative value: 56%; LHR + : 84%; LHR-: 17%). Chronic response was not observed in 100% vs. 0%, *p* = 0.004 patients with spleen TE ≥ 74KPa and SHVDI > 0.6. The combination of both measurements for predicting poor chronic response to beta-blockers has an AUC of 0.8 (0.7–0.9) (Figure 5).

## Discussion

The hemodynamic response to beta-blockers measured by HVPG is an invasive procedure that is not available in most centers (5–7). So far, we lack good tools that allow us to non-invasively assess whether our patient is going to be a beta-blocker responder or not. Therefore, the role of non-invasive measurements of portal hypertension has to be elucidated for this purpose.

In our study, we evaluated the correlation between the different invasive measurements and HVPG. As expected, liver TE, spleen TE, liver ARFI, and spleen ARFI showed a moderate correlation with HVPG. The correlation between HVPG and elastographic techniques has been widely validated in multiple studies (14–17). Among the Doppler ultrasonography measurements, the measurements that showed a moderate-weak correlation with HVPG were portal vein diameter and damping index. In a previous study, the damping index showed a strong correlation with HVPG (Spearman coefficient: 0.8, *p* < 0.01) (33); in our study, the correlation was not so strong but reached statistical significance, perhaps due as well to the small sample size. Moreover, we could not find any correlation between hepatic vein contrast transit and HVPG. Hepatic vein transit time decreases as long as the disease progresses and the severity of portal hypertension increases due to intrahepatic shunts (34). In our study, we included patients who already had esophagogastric varices. In this subgroup of patients portal hypertension is more severe and they probably already have more intrahepatic shunts so probably the hepatic vein transit time is lower.

Acute response to beta-blockers was positive in 68.3% of the patients, and chronic response to beta-blockers was observed in 36.7%, which is consistent with previously published data (5, 7). Considering that carvedilol was used mainly to evaluate chronic response (only 4 patients had propranolol as secondary prophylaxis), our results in chronic response were slightly lower. In any case, the confidence interval for the response reaches 55%, so these changes can be explained by the variability of the specific sample. According to previous studies (6, 29), we did not find any baseline clinical characteristics or laboratory tests related to acute or chronic response to beta-blockers. As in the study by Kim et al. baseline spleen or liver ARFI were not related either to acute or chronic response to beta-blockers (29). In fact, the only baseline non-invasive measurements that were statistically related to acute or chronic response to beta-blockers were spleen TE [non-acute responders 75 KPa (*SD*: 0) vs. acute responders 58.4 KPa (*SD*: 23), *p* = 0.04; non-chronic responders 73.2 KPa (*SD*: 5.5) vs. chronic responders 58.1 KPa (*SD*: 21.4), *p* = 0.02] and damping index [non-acute responders 0.96 (0.8) vs. acute responders 0.44 (0.4), *p* = 0.01; non-chronic responders 0.8 (0.7) vs. chronic responders 0.4 (0.4), *p* = 0.04]. These results suggest that patients with significantly more severe portal hypertension are poor responders to beta-blockers. In fact, elevated spleen TE and elevated damping index had good accuracy to predict poor acute or chronic response to beta-blockers. We evaluated the best cutoff points of both measurements to predict poor acute or chronic response to beta-blockers. A spleen TE ≥ 74 KPa had a high negative predictive value (acute response 100%, chronic response 71%), suggesting that patients with a spleen TE below 74 have a high probability of response to beta-blockers. In contrast, the damping index > 0.6 had a high negative predictive value in acute response to beta-blockers (87%), but it was not so high in chronic response (56%). Thus, the combination of both indices selected a group of patients with a significantly poor response to acute or chronic response to beta-blockers. This population represented 8 (19.5%) patients evaluated for acute response and 10 (33.3%) patients evaluated for chronic response. Thus, as we have previously mentioned, patients with a higher degree of basal portal hypertension are probably worse acute and chronic responders. This is confirmed by the fact that acute and chronic responders had lower HVPG. This probably explains why we have been able to detect how non-invasive baseline measurements of portal hypertension were related to poor acute or chronic response to beta-blockers.

Despite the fact that we observed that values of spleen TE and spleen ARFI decreased after acute and chronic response to beta-blockers, we were not able to demonstrate that changes in the values after the acute or chronic evaluation with respect to baseline determinations were related to them. Kim et al. (29) showed that changes in spleen ARFI had a good ability to predict chronic response to beta-blockers. What is more, a recent study with a small sample size (*n* = 20) also determined that the changes in spleen TE were capable of predicting chronic response. We hypothesize that the reasons for the absence of results in our study are diverse. In the case of liver elastography, this was reasonably expected given the fact that it is unlikely to observe changes in liver fibrosis measured by elastographic techniques after evaluating acute or chronic response to beta-blockers. In the case of spleen elastography, perhaps, our study has not allowed us to observe changes given the small sample size. Besides, the fact of not having the spleen-specific probe that measures values until 100 KPa has also contributed to the lack of results. More studies are probably needed to confirm these results.

The main limitation of our study is that the sample size is small. However, to the best of our knowledge, this is the first study that evaluates non-invasive measurements for predicting acute response to beta-blockers. What is more, it is a prospective study in which all the non-invasive measurements that were intended to be studied were performed before and after the acute and chronic responses.

## Conclusion

Spleen TE and damping index can identify a subgroup of patients with poor acute or chronic response to beta-blockers. Changes in non-invasive measurements were not able to identify poor responders.

## Data Availability Statement

The raw data supporting the conclusions of this article will be made available by the authors, without undue reservation.

## Ethics Statement

The studies involving human participants were reviewed and approved by Comité de Ética del Hospital Universitario Puerta de Hierro. The patients/participants provided their written informed consent to participate in this study.

## Author Contributions

JR, EL, and JC: study concept and design. EL, CP, MC, ML, CF, CC, TF, JM, JA, NP, MT, IH, and ES: data acquisition. EL and JC: data analysis and interpretation. EL: drafting of the manuscript and statistical analysis. JC: obtain funding. All authors contributed to the article and approved the submitted version and critical revision of the manuscript for important intellectual content.

## Conflict of Interest

JC reports consultancy and lecture fees from Abbvie, Gilead Sciences, MSD. The remaining authors declare that the research was conducted in the absence of any commercial or financial relationships that could be construed as a potential conflict of interest.

## Publisher’s Note

All claims expressed in this article are solely those of the authors and do not necessarily represent those of their affiliated organizations, or those of the publisher, the editors and the reviewers. Any product that may be evaluated in this article, or claim that may be made by its manufacturer, is not guaranteed or endorsed by the publisher.

## Abbreviations

HVPG: hepatic venous pressure gradient
CSPH: clinical significant portal hypertension
TE: transient elastography
ARFI: acoustic radiation force impulse
FSHP: free suprahepatic pressure
WSHP: wedge suprahepatic pressure
AUC: area under the curve.
