# Supplementary material for: Spleen Transient Elastography and Damping Index Identify a Subgroup of Patients Without an Acute or Chronic Response to Beta-Blockers
Source: Front Med (Lausanne). 2022 Jun 22;9:900073. doi: 10.3389/fmed.2022.900073 (PMC9258685; doi:10.3389/fmed.2022.900073)
Supplement: Supplementary file 1 [file Table_1.docx]

Supplementary Material

**Table 1 Suppl.** Correlation between HVPG, elastographic and ultrasound parameters.

|  | **Pearson o Spearman correlation coefficient** | **p** |
| --- | --- | --- |
| **Hepatic TE** | 0.5 | 0.01 |
| **Spleen TE** | 0.3 | 0.04 |
| **Hepatic ARFI** | 0.4 | 0.02 |
| **Spleen ARFI** | 0.5 | <0.01 |
| **Portal vein diameter** | 0.4 | 0.02 |
| **Portal vein speed** | -0.01 | 0.9 |
| **Hepatic artery speed** | 0.3 | 0.2 |
| **Resistance index hepatic artery** | -0.1 | 0.5 |
| **Spleen diameter** | -0.03 | 0.8 |
| **Hepatic vein transit time** | -0.1 | 0.5 |
| **Damping index** | 0.2 | 0.04 |

**Table 2 Suppl.** Univariate analysis of baseline characteristics between acute and chronic responders and non responders to beta-blockers.

|  | **Non acute response to beta-blockers**  **n= 13** | **Acute response to beta-blockers n=28** | **p** | **Non chronic response to beta-blockers**  **n=19** | **Chronic response to beta-blockers**  **n=11** | **p** |
| --- | --- | --- | --- | --- | --- | --- |
| **Age (y)** | 59.0 (10.2) | 56.0 (7.1) | 0.3 | 56.5 (9.1) | 57.8 (5.3) | 0.6 |
| **Sex (M:F)(%)** | 100:0 | 78.6:21.4 | 0.1 | 84.2:15.8 | 90.9:9.1 | 0.9 |
| **Etiology**  Hepatitis C virus  Alcohol  Autoinmune  Non alcoholic fatty liver disease  Other | 25  33.3  16.7  25  0 | 28.6  46.4  7.1  7.1  10.7 | 0.5 | 26.3  42.1  10.5  15.8  5.3 | 27.3  36.4  0  18.2  18.1 | 0.5 |
| **Child Pugh**  A  B  C | 66.7  16.7  16.7 | 85.7  14.3  0 | 0.1 | 68.4  26.3  5.3 | 90.9  9.1  0 | 0.5 |
| **MELD** | 11.8 (5.1) | 9.9 (3.3) | 0.2 | 10.4 (3.0) | 10.3 (2.2) | 0.9 |
| **Laboratory test**  Leucocites (cels*10^9^L)  Hemoglobine (mg/dl)  Platelets (cels*10^9^L)  Creatinine (mg/dl)  Sodium (mg/dl)  Potasium (mg/dl)  Bilirrubine (mg/dl)  Albumine (mg/dl)  ALT (U/L)  AST (U/L)  Alkaline phosphatase (U/L)  GGT (U/L)  INR | 5.0 (2.2)  13.0 (2.4)  96.8 (29.7)  0.8 (0.2)  139.0 (4.6)  4.3 (0.7)  1.8(1.4)  3.8 (0.7)  37.0 (44.6)  48.3 (46.3)  174.2 (187.4)  124.4 (140.1)  1.4 (0.5) | 6.5 (3.2)  12.8 (3.2)  118.6 (54.3)  0.8 (0.2)  140.1 (3.0)  4.3 (0.4)  1.4 (1.8)  3.9 (0.6)  43.0 (27.3)  52.5 (30.6)  115.6(54.4)  121.9 (78.0)  1.3 (0.2) | 0.2  0.9  0.2  0.7  0.4  0.9  0.6  0.5  0.6  0.7  0.1  0.9  0.2 | 6.2 (2.8)  12.6 (2.3)  118.5 (42.8)  0.8 (0.2)  140 (3.7)  4.4 (0.5)  1.5 (1.1)  3.8 (0.6)  35.9 (37.9)  47.9 (38.2)  159.8 (151.1)  120.2 (117.7)  1.3 (0.2) | 6.3 (2.8)  13.5 (1.2)  113 (49.5)  0.8 (0.2)  139.7 (3.6)  4.0 (0.5)  1.2 (0.4)  4 (0.7)  45.5 (27.5)  53.3 (35.0)  114.6 (55.9)  120.2 (75.1)  1.3 (0.2) | 0.9  0.3  0.8  0.7  0.8  0.1  0.3  0.3  0.5  0.7  0.40.9  0.8 |

*Quantitative data are given by mean and standard deviation, qualitative data are given by percentage. ** ALT alanine aminotransferase AST aspartate aminotransferase GGT gamma glutamil transferase, INR International normalized ratio.

**Table 3 Suppl.** Follow up non invasive measurements after acute response to betablockers.

|  | **Non acute response to betablockers**  **n=13** | **Acute response to betablockers**  **n= 28** | **p** |
| --- | --- | --- | --- |
| **Change in liver TE (KPa)** | - 2.1 (7.5) | -1.6 (13.7) | 0.9 |
| **Change in spleen TE (KPa)** | 0 (0) | 0.5 (11.5) | 0.9 |
| **Change in liver ARFI (m/s)** | 0.3 (0.5) | 0.1 (0.8) | 0.4 |
| **Change in spleen ARFI (m/s)** | 0.1 (0.3) | 0.1 (0.3) | 0.7 |
| **Change in portal vein diameter (mm)** | 0.2 (1.8) | -0.2 (3.7) | 0.8 |
| **Change in portal vein speed (cm/s)** | -0.4 (4.9) | 0.9 (4.9) | 0.5 |
| **Change in hepatic artery speed (cm/s)** | -7.9 (14.5) | 4.2 (35.9) | 0.4 |
| **Change in resistance index hepatic artery** | 0.03 (0.1) | -0.01 (0.05) | 0.1 |
| **Change in spleen diameter (cm)** | -0.2 (0.5) | 0 (0.7) | 0.5 |
| **Change in hepatic transit time (s)** | -3.4 (4.9) | -1.8 (6.9) | 0.5 |
| **Change in damping index** | -0.02 (2.6) | 0.95 (7.6) | 0.2 |

**Table 4 Suppl.** Follow up non-invasive measurements after chronic response to betablockers.

|  | **Non chronic response to betablockers**  **n=19** | **Chronic response to betablockers**  **n= 11** | **p** |
| --- | --- | --- | --- |
| **Change in liver TE (KPa)** | 6.2 (17.6) | 3.45 (17.6) | 0.8 |
| **Change in spleen TE (KPa)** | 0 (0) | -11.1 (0) | 0.9 |
| **Change in liver ARFI (m/s)** | 0.1 (0.5) | 0.1 (0.7) | 0.9 |
| **Change in spleen ARFI (m/s)** | 0.1 (0.3) | -0.02 (0.2) | 0.2 |
| **Change in portal vein diameter (mm)** | -0.1 (2.1) | -0.15 (2.3) | 0.9 |
| **Change in portal vein speed (cm/s)** | 0.5 (6.5) | 2.12 (3.9) | 0.5 |
| **Change in hepatic artery speed (cm/s)** | 11.2 (22.9) | 27.3 (41.9) | 0.4 |
| **Change in resistance index hepatic artery** | 0.1 (0.3) | -0.01 (0.06) | 0.5 |
| **Change in spleen diameter (cm)** | -0.03 (0.8) | -0.2 (1.5) | 0.8 |
| **Change in hepatic transit time (s)** | -5.6 (8.3) | -4.2 (8.5) | 0.7 |
| **Change in damping index** | 0.4 (0.7) | 0.1(0.4) | 0.2 |
